# Etiology and incidence facial fractures in children and adults

**DOI:** 10.1016/S1808-8694(15)30061-6

**Published:** 2015-10-19

**Authors:** Jair Cortez Montovani, Lígia Maria Pirani de Campos, Marina Ayabe Gomes, Vinícius Rodrigues Silva de Moraes, Fabricio Dominici Ferreira, Emanuel Araújo Nogueira

**Affiliations:** a Associate Professor.; b 6th Year Medical Student.; c 6th Year Medical Student.; d 6th Year, Medical Student.; e MD, 3rd Year Otorhinolaryngology Resident.; f MD, Otorhinolaryngologist.

**Keywords:** Facial fracture, Trauma, Urban violence

## Abstract

Facial trauma has presented an increasing occurrence in the last four decades, due especially to the growth of accidents with automobiles as well as to the urban violence. Both of which continue being the main cause of such traumas. **Aim:** To evaluate the features of the population victim of facial trauma as to gender, age, occupation, origin, type of fracture and its cause. **Design study:** retrospective clinical with transversal cohort. **Material and Method:** Retrospective study consulting hospital registers of 513 patients victms of the facial trauma. **Results:** There was a higher incidence of facial trauma on men (84,9%), white (82,7) and with an average age of 29. Regarding occupation, the trauma was mostly occurred to students (16,6%) and Masons (11,2%). The jaw was the most affected place (35%), followed by zygoma (24%) and by the nose (23%), though most patients presented a single facial fracture (82,5%). Among the causes, accidents with automobiles (28,3%), aggressions (21%) and accidental fall s (19,5%) were the most common. **Conclusions:** Accidents with automobiles continue being the main cause of facial trauma, especially of multiple factures due to the great transmission of kinetic energy.

## INTRODUCTION

In the last four decades facial trauma has become an unavoidable theme among physicians due to increased frequency as a result of the growing incidence of motor vehicle accidents and urban violence^1^, ^2^, ^3^, ^4^, ^5^, ^6^. Facial skin and bone are extremely exposed to such trauma due to their anterior location. Skin is thin and elastic, subcutaneous tissue is delicate, muscles are superficial and there is extensive vascularization and innervation. When compressed between bone and external trauma forces, soft tissue may present a variety of injuries (cuts, laceration, hemorrhage, hematomas, etc.) adding to the harmful effects of bone fractures^7^, ^8^.

Facial trauma has a heterogeneous etiology and the predominance of one or another factor is due to characteristics of the population under study (age, gender, social status, urban and residential sites)^9^, ^10^, ^11^, ^12^. In certain regions of our country and in parts of Europe bicycles are widely used for leisure or transport, increasing the possibility of accidents with this vehicle. Facial fractures result from games and child play in children and falls at home in the elderly^13^, ^14^, ^15^, ^16^, ^17^, ^18^. The most common causes for young people up to the fourth decade of life include motor vehicle accidents, physical aggression and sports trauma^19^, ^20^, ^21^, ^22^.

At present the association between alcohol and drug use, driving and urban violence leads to increasingly complex facial trauma^25^. It is no coincidence that most of these injuries occur during weekends when parties, bars, and other similar activities favor drug and alcohol abuse for leisure and fun.

Understanding the gravity of this situation, society attempts to organize itself to face this authentic war. Educational preventive campaigns together with strict laws, particularly for traffic infractions, attempt to change the present frightening scenario of motor vehicle accidents and urban violence^26^, ^27^. For some authors the introduction of safety devices, including compulsory use of seat belts, air bags and side protection bars begin to reduce if not the rates, at least the complexity of facial fractures^27^, ^28^. Cavington et al. (1994) show that seat belt adoption in the USA during the past 10 years reduced the incidence of multiple facial fractures, particularly zygomatic bone fractures, from 46.3% and 80.6% to 20.1% and 50%^28^, ^29^. Preliminary data in Brazil show that the incidence of traffic accidents is falling. Between 1991 and 2000, there was a 10.4% reduction in the proportion of deaths per traffic accident as part of the total number of accidents, which now stands at 25%, whereas the proportion of homicides increased 27.2%, reaching 38.3% of the total number of accidents.

Data from the Brazilian Association of Traffic Departments in four state capitals show that 27.2% of traffic accident victims had blood alcohol content above the 0.6 g/L legal limit.

Such violence raises questions about the capability of health units to offer adequate emergency care to victims.

The common reality in most emergency units is that almost always there are no teams prepared for this kind of care; when such care is provided, it is chaotic and fragmented. Usually during the first moments there is confusion and competition between health professionals about who should see these patients, particularly between specialists with common areas of expertise^31^. Not uncommonly this lack of decision is aggravated by the absence of a classification of cranial and facial trauma, causing difficulties for rational and integrated efforts between medical specialties involved in the care of trauma patients^32^.

The aim of this paper is to describe the experience of the Botucatu Medical College Clinical Hospital (Hospital das Clinicas da Faculdade de Medicina de Botucatu), Sao Paulo State, in the care of facial fracture patients. Priority is given to epidemiological data (age, gender, profession, origin), etiology of fractures, fracture site (lower jaw, zygoma, upper jaw, frontal and nasal bones), fracture type (simple, multiple) and the association with the use of drugs (alcohol).

## MATERIAL AND METHODS

A retrospective, non-randomized study was made of 513 patients diagnosed with facial fractures at the Botucatu Medical College Clinical Hospital (Hospital das Clínicas da Faculdade de Medicina de Botucatu) ENT and Head and Neck Surgery Department between 1991 and 2004).

The data collection protocol included: age, gender, city of origin, fracture site, type of fracture, etiology of the trauma, drug abuse and seat belt use. EPI - INFO 6.04 was used to analyze data.

The fracture etiology was classified as: motor vehicle accidents (cars, motorbikes, and trucks), bicycles, physical violence, accidental falls, sports accidents, accidents with animals and other causes.

Facial fractures were classified as: mandibular, zygomatic, maxillary, nasal and frontal fractures. A fracture was simple if only a single bone was involved, multiple if two or more bones were fracture and associated when other bones in the body were involved.

## RESULTS

**Graph 1:**
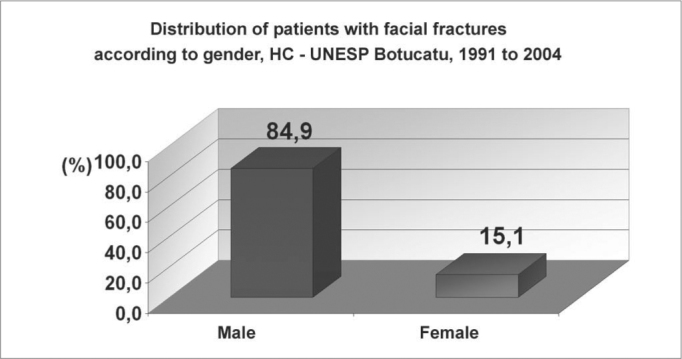
Distribution of patients with facial fractures according to gender, HC - UNESP Botucatu, 1991 to 2004

**Graph 2:**
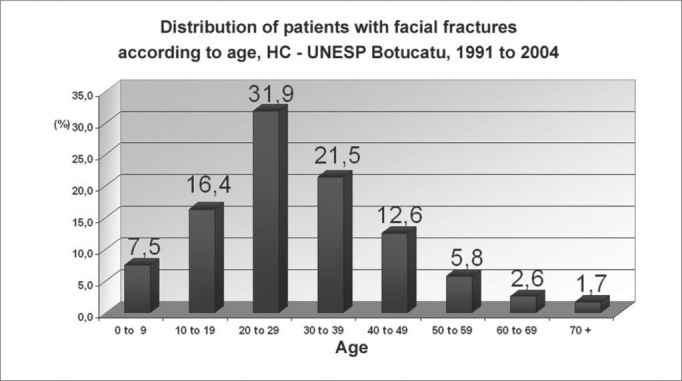
Distribution of patients with facial fractures according to age, HC - UNESP Botucatu, 1991 to 2004

**Graph 3:**
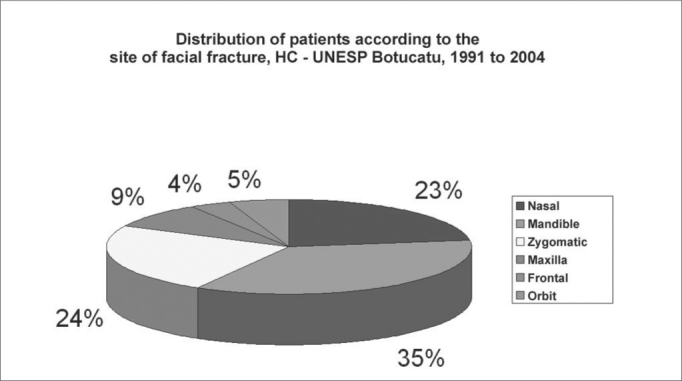
Distribution of patients according to the site of facial fracture, HC - UNESP Botucatu, 1991 to 2004

Facial fractures were diagnosed in 513 patients, of which 77 were women (15.1%) and 436 were men (84.9%) (Graph 1). The highest incidence was in the 20 to 29 years age group; approximately two-thirds of the fractures (69.8%) were in the 11 to 39 years age group (Graph 2). There were 565 fractures, an average 1.1 per victim. Mandibular fractures were the most common type (35%), followed by zygoma fractures (24%) and nasal fractures (23%) (Graph 3).

The etiology of fractures is shown on Tables 1 and 2: 169 (32.94%) were motor vehicle accidents (cars, trucks, buses, motorbikes), of which 13 were pedestrian crashes; 129 (25.1%) were due to physical violence, 89 (17.2%) where the result of falls, 47 (9.2%) were bicycle accidents, 27 (5.3%) were sports injuries, 25 (4.9%) were accidents with animals and 7,4% were due to other injuries.

**Table 1:** Distribution of patients with facial fractures according to age and etiology of trauma.

Etiology									
Age	AM	B	AT	Q	AG	E	AN	O	total
0-9	3	4	1	15	2	1	3	3	32(6,23)
10- 19	27	10	3	10	9	7	6	6	78(15,2)
20-29	60	17	1	19	38	15	8	7	167(32,55)
30-39	37	6	6	18	31	2	3	6	114(22,22)
40-49	13	6	0	8	17	1	2	13	62(12,08)
50-59	10	4	2	8	6	1	0	2	33(6,43)
60-69	4	0	0	3	4	0	2	1	16(2,26)
70 +	2	0	0	8	0	0	1	0	11(2,14)
Total %	156(30,5)	47(9,2)	13(2,5)	89(17,2)	118(23)	27(5,3)	25(4,9)	38(7,4)	513

**Table 2:** Distribution of patients according to etiology and fracture site.

	Nasal	Zygoma	Mandibula	Maxila	Frontal	Orbit	Total	%
AM	31	42	53	19	9	12	166	29,4
B	6	15	23	5	1	2	52	9,2
AT	2	2	8	2	1	1	16	2,8
Q	27	20	35	9	3	5	99	17,5
AG	34	30*1	54*3	7	5	3	137	24,2
E	10	13	5	0	2	2	32	5,7
A	6	7	14	3	1	2	33	5,8
0	4	9	10	3	0	4	30	5,4
Total	120	139	205	48	22	31	565	100,00

AM: motor vehicle accident, B: bicycle, AT: pedestrian crash, Q: accidental fall, AG: aggression, E: sports, AN: accidents with animals, O: other causes.

The most frequent etiology of fractures in children (0 to 9 years) and adults over 60 years was falls (46.8% and 40.7% respectively). Motor vehicle accidents were the main cause of facial trauma in the 20 to 29 year age group (35.9% of cases).

**Graph 4:**
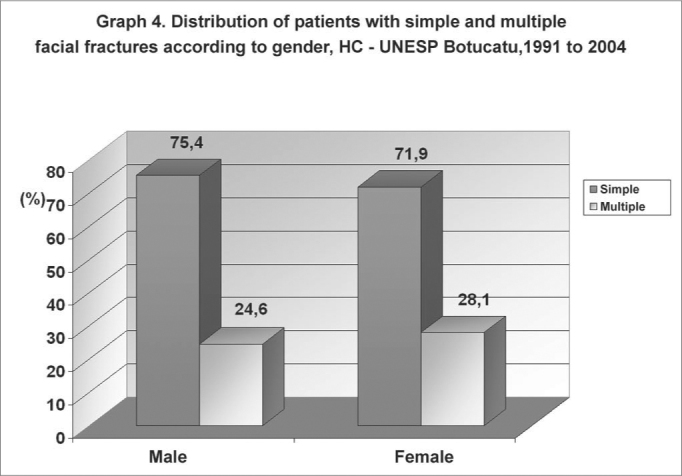
Distribution of patients with simple and multiple facial fractures according to gender, HC - UNESP Botucatu, 1991 to 2004

**Graph 5:**
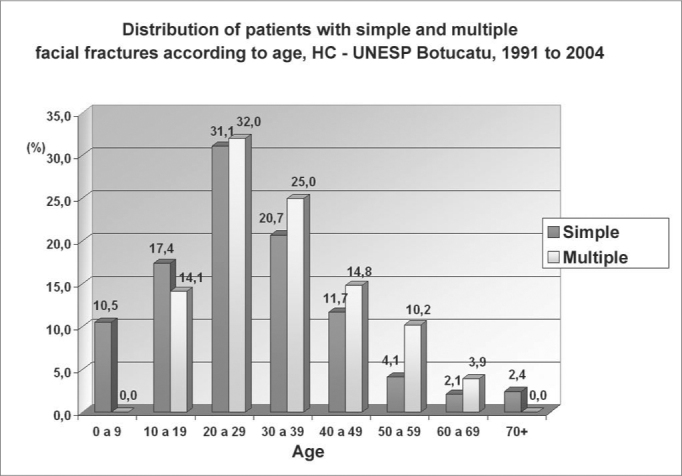
Distribution of patients with simple and multiple facial fractures according to age, HC - UNESP Botucatu, 1991 to 2004

There were 371 (72.3%) simple facial fractures (a single fracture in the face) and 142 (27.6%) multiple facial fractures (Graphs 4 and 5). The causes of simple facial fractures were: 24.6% due to motor vehicle accidents, 22.6% resulting from physical violence, 20.1% due to falls, 9% from bicycle accidents and 9% related to sports. The causes of multiple facial fractures were: 34% due to motor vehicle accidents, 17% from falls, 14.9% resulting from physical violence and 9.6% from accidents with animals. Different from simple fractures, multiple fractures involved mostly men, particularly in the 20 to 49 year age group.

Motor vehicle accidents (25.6%) and physical violence (24.4%) were the main cause of nasal fractures. Motor vehicle accidents (27.7%) followed by falls and physical violence, both at 21.2%, were the main causes of mandibular fractures. Motor vehicle accidents were the main cause of zygoma fractures (32.6%).

38% of patients involved in motor vehicle accidents and in 58% of physical violence victims in the 15 to 39 year age group declared that they had taken alcohol minutes or hours before the accident. 45% of patients were not using the seat belt.

## DISCUSSION

A significant finding in our study was the marked difference in the incidence of facial trauma in men and women (84.9% and 15.1% respectively). These numbers are similar to those of Sherer et al.^11^ in an analysis of 788 patients where 80.7% were men and 19.3% were women. The increased incidence in men may be explained by the fact that men are more frequent drivers, particularly on highways. Men also tend practice physical contact sports (soccer, basketball, martial arts, etc), frequently go to “bars” as their social activity and consequently tend to use drugs, including alcohol, before driving. However, in the last three decades, there has been a growing incidence of trauma in women, mostly before 40 years of age^26^, ^27^. This is due to a change in female behaviors in society, including a higher number of women drivers, an association between alcohol and driving, a larger number of women working out of home and the practice of sports as leisure and health activities, including sports involving physical contact such as soccer, basketball and martial arts^26^, ^27^.

Other associated factors such as drug abuse, family frailty and fragmentation and unemployment were not adequately assessed in our emergency hospital data. Many patients arrived hours or even days following trauma and often omitted information on the use of alcohol and details of the accident or aggression. Of concern was the information that below 30 years of age 38% of motor vehicle accident patients and 68% of physical violence victims had consumed alcohol. Our observations confirm those reported by Mac Dade et al. (1982)^25^, where psychosocial factors leading to violence in contemporary urban society and social, economic and emotional conflict in young people are given as the causes of increased external violence^11^, ^19^, ^25^. It is understandable that violence may occur more frequently among young people as a result of their disquiet and disobedience of social norms, including traffic laws, influenced by extremely rapid behavioral and moral changes.

In a 1957 study of 1,000 accidents with 2,253 victims, Braustein1 reported that 72.1% of cranio-facial trauma was due to accidents with vehicles. In 1961 Hagan and Huelke23 reported that 55.8% of mandibular fractures were due to motor vehicle accidents, 17% were caused by physical violence and 14% were due to other causes. In 1968 Rowe and Killey^24^ reported that 11.6% of mandibular fractures were due to motor vehicle accidents, 15.8% were motorbike accidents, 18% were caused by physical violence and 12% resulted from accidental falls.

Other population characteristics, such as living in urban or rural areas and rich or poor neighborhoods, social and economic status and education influenced the etiopathogenesis and severity of facial trauma^4^, ^9^, ^10^, ^11^. In 1967 Shultz^3^ noted that in poor neighborhoods, physical violence was the most frequent cause (35.1%), followed by motor vehicle accidents (26.3%) and sports injuries (12.2%). In richer neighborhoods, the main cause was motor vehicle accidents. Our study shows similar data, with physical violence very common in lower income groups and in people with professions such as bricklayers and wall painters and the unemployed. In these groups nearly always the “bar” was the option for leisure.

Fractures were less frequent in children and adults over 60 years of age. Posnick^18^ and Lucht^13^ noted that the lower incidence of facial trauma in these age groups is due to family care, more time spent at home, surveillance of children, characteristics of the elderly where there is less social and sports activity, longer stay at home and the need for a companion when going out^13^, ^14^, ^15^, ^16^, ^17^, ^18^. In these age groups trauma is usually simple fractures related to household accidents such as slipping, falling from stairs and child play.

The majority of sports-related facial injuries occurred in collective sports, as expected mostly soccer, as the sport most frequently practiced in this country. However some of these facial injuries could be considered true aggression, as punches, kicks, elbowing, heading etc. were described as nearly always intentional. This is not new to those who watch these sports which are frequently shown in the media. The majority are simple fractures, most commonly nasal and zygoma fractures.

Many accidental fractures were probably caused by parental abuse (father, mother, husband, and son). In some of these cases, by the end of treatment enough trust had been established between the physician and the victim, many of which would then reveal the reason for omitting the cause of injury: shame, fear of future aggression, having to return to the same house in which the aggressor lived, fear of divorce due to financial dependence and the feeling that aggressors went unpunished^11^. Again, there was a frequent association with drug and alcohol abuse^4^, ^5^, ^19^, ^25^.

Our data concerning the site of fractures are different from literature. The incidence in decreasing order was: mandibular, nasal zygoma, maxillary and frontal fractures. An explanation for this apparent inversion at our unit might be under notification of nasal trauma which are corrected in local emergency units and the lack of nasal fracture diagnosis in children. Less marked projection of the nasal pyramid in children, incomplete nasal bone ossification and lack of examiner experience may lead to errors in the clinical and image interpretation of these fractures^1^, ^2^, ^3^, ^4^, ^24^. Almost always the physician is concerned with pain, nasal bleeding and swelling and never with observation of septal deviation and nasal pyramids^14^, ^18^. Therefore children and their caretakers are misinformed and they almost never return for a second look.

A similar situation can occur in lower orbital wall fractures with small bone misplacement that go unseen in the first few hours following trauma because of eye lid swelling and imaging only with plain cranial radiography. In this situation the fracture may be difficult to see due to overlapping of anatomical structures (orbit wall and temporal bone)^12^, ^23^. According to Busuito et al. (1986) and Sherer et al. (1989) these failures in diagnosis do not occur with other facial fractures (mandibular and maxillary fractures), mostly caused by motor vehicle accidents and physical violence. In these cases, hematomas, the difficulty to open the patient’s mouth, hemorrhage, esthetic deformity and associated injuries (cranio-facial and other parts of the body) and legal issues foster careful clinical observation and diagnosis.

For these reasons, mandibular fractures were apparently the most frequent at our unit, almost always one of three etiologies: motor vehicle accidents, falls and aggression. The majority of our patients had partially or fully edentulous lower jaws, periodontitis, dental caries and teeth at the fracture site^9^, ^10^. We therefore found a high rate of soft tissue infection and loss of teeth (12%), almost always aggravated by the excessive mobility of fractured bone and not uncommonly, by delayed referral to our hospital.

The most common causes of maxillary fractures (Le Fort), except for the Le Fort I, were motor vehicle accidents. Many of these fractures were complex and associated with cranioencephalic complications such as pneumoencephalus, meningitis, basal skull fractures and CSF fistula. Le Fort I type fractures were apparently rare in our unit, but we believe that they might have been under notified, especially in dental trauma with dental weakening and loss. These cases are usually referred to the dental surgeon or patients and family members may never seek medical help. In children with dental trauma we commonly see a lack of family concern for dental and soft tissue (alveolar) treatment in children with deciduous teeth due to the possibilities of secondary dentition. They do not know that such trauma may lead to changes in dental germ growth and other associated complications.

It is our opinion that all facial fractures, including nasal fractures, should be treated surgically under sedation or general anesthesia in the operating room either immediately before edema becomes marked or between the 3rd and 7th day after edema has disappeared. Other options such as correction with the patient awake, therefore feeling pain and unable to cooperate adequately, induce failure and may require a second or third surgical procedure^11^, ^12^, ^18^. In our experience we have seen a large number of pyramid and nasal septum deviation due to previous trauma treated conservatively or with inadequate surgical correction in hospital emergency wards.

Zygoma fractures should be treated using a direct surgical access route (subcilliary or transconjunctival approach) to reposition the fragments. In a few cases we used a hook (Ginetest) to elevate bone fragments as an ancillary method for zygomatic arch cases. Cases presenting diplopia and other complications (enophthalmos and optic nerve compression) are preferably operated as soon as possible due to the possibility of entrapment and necrosis of external orbital muscles, nerve compression and amaurosis. Unfortunately this is not always possible as delays occur due to diagnostic errors or difficulties in referring a patient from one hospital to another. We use porous polyethylene plastic implants, cartilage or parietal bone external lamina to reconstruct the orbit floor or cavity, as described in literature.

Missing teeth, upper or lower dental prostheses, tooth decay, infected teeth and teeth located within fractures added difficulties to mandibular fracture correction.

Fractures of the mandibular condyle were treated conservatively and/or with intermaxillary fixation for three weeks plus ostheosynthesis. Steel wires were used in the not so distant past to treat edentulous patients with multiple or comminuted fractures or infection; in the past ten years we have adopted miniplates. Our results show that results using steel wires and miniplates are similar, both for zygomatic and mandibular fractures; steel wires therefore are a good solution particularly due to practicality and low cost.

Frontal sinus fractures were uncommon in our study, usually located in the external wall and in general uncomplicated except for bone depression. As in maxillary fractures there were numerous complications in injuries of the internal wall, such as pneumoencephalus, meningitis and CSF fistulae. Treatment was always surgical, but in men a surgical incision below the eyebrow was preferred as opposed to the bicoronal incision used in the 70s and 80s. The latter incision was only used in fractures of the cribriform plate of the ethmoid bone associated with CSF fistulae (3 patients) due to the need for muscle flap rotation to protect the dura mater. The bicoronal incision can compromise male esthetics, even accelerating baldness. Reconstruction with silastic^(4)^ and porous polyethylene^(3)^ was used for comminuted sinus fractures or when there was loss of tissue. Porous polyethylene extrusion was seen in one case, which was substituted by a parietal bone osteocutaneous flap. In some cases the need for inserting a catheter into the nasofrontal duct is still debated in literature. We believe the best strategy is debridement and curettage of necrotic mucosa and, if necessary, insertion of a catheter into the nasofrontal duct (2 patients) and long term antibiotics (8 weeks). Complications such as mucoceles may be treated in a second surgical procedure with nasal endoscopic surgery.

## CONCLUSION

Facial fractures are common and result mostly from motor vehicle accidents, physical violence and falls. The highest incidence is in young male patients (15 to 40 years of age) and not uncommonly is associated with other fractures and potentially severe injuries. Facial bones most frequently involved were the mandible, the nasal bone and the zygoma. These numbers, however, may be biased due to under notification of nasal fractures. Simple or low complexity fractures correlate with physical aggression whereas high complexity fractures are correlated with motor vehicle accidents. A significant proportion of these accidents are associated with drug abuse, speeding and disregard for the use of seat belts. Increased hospital stay may be due to delays in care, difficulties in referring patients (due to the lack of hospital beds at our unit, among other reasons), the complexity of injuries and the need for neurological observation and care in many cases. Complications depended on the fracture and may be summarized as follows: infection, dental malocclusion, loss of teeth, diplopia and other changes in vision, rhinolaterality, rhinosclerosis and nasal obstruction.
